# Low-Dose Radiation Can Cause Epigenetic Alterations Associated With Impairments in Both Male and Female Reproductive Cells

**DOI:** 10.3389/fgene.2021.710143

**Published:** 2021-08-02

**Authors:** Chi Tim Leung, Yi Yang, Kwan Ngok Yu, Nathan Tam, Ting Fung Chan, Xiao Lin, Richard Yuen Chong Kong, Jill Man Ying Chiu, Alice Sze Tsai Wong, Wing Yee Lui, Karen Wing Yee Yuen, Keng Po Lai, Rudolf Shiu Sun Wu

**Affiliations:** ^1^Hong Kong Branch of the Southern Marine Science and Engineering Guangdong Laboratory, Hong Kong, China; ^2^Department of Chemistry, City University of Hong Kong, Kowloon Tong, Hong Kong; ^3^School of Biological Sciences, The University of Hong Kong, Pok Fu Lam, Hong Kong; ^4^State Key Laboratory of Marine Pollution, City University of Hong Kong, Kowloon Tong, Hong Kong; ^5^Department of Physics, City University of Hong Kong, Kowloon Tong, Hong Kong; ^6^School of Life Sciences, Hong Kong Bioinformatics Centre, The Chinese University of Hong Kong, Shatin, Hong Kong; ^7^Department of Biology, Hong Kong Baptist University, Kowloon Tsai, Hong Kong; ^8^Laboratory of Environmental Pollution and Integrative Omics, Guilin Medical University, Guilin, China; ^9^Department of Science and Environmental Studies, The Education University of Hong Kong, Tai Po, Hong Kong

**Keywords:** environmental radiation, epigenetic, reproductive impairments, testicular, ovarian

## Abstract

Humans are regularly and continuously exposed to ionizing radiation from both natural and artificial sources. Cumulating evidence shows adverse effects of ionizing radiation on both male and female reproductive systems, including reduction of testis weight and sperm count and reduction of female germ cells and premature ovarian failure. While most of the observed effects were caused by DNA damage and disturbance of DNA repairment, ionizing radiation may also alter DNA methylation, histone, and chromatin modification, leading to epigenetic changes and transgenerational effects. However, the molecular mechanisms underlying the epigenetic changes and transgenerational reproductive impairment induced by low-dose radiation remain largely unknown. In this study, two different types of human ovarian cells and two different types of testicular cells were exposed to low dose of ionizing radiation, followed by bioinformatics analysis (including gene ontology functional analysis and Ingenuity Pathway Analysis), to unravel and compare epigenetic effects and pathway changes in male and female reproductive cells induced by ionizing radiation. Our findings showed that the radiation could alter the expression of gene cluster related to DNA damage responses through the control of MYC. Furthermore, ionizing radiation could lead to gender-specific reproductive impairment through deregulation of different gene networks. More importantly, the observed epigenetic modifications induced by ionizing radiation are mediated through the alteration of chromatin remodeling and telomere function. This study, for the first time, demonstrated that ionizing radiation may alter the epigenome of germ cells, leading to transgenerational reproductive impairments, and correspondingly call for research in this new emerging area which remains almost unknown.

## Introduction

Humans are regularly and continuously exposed to ionizing radiation from both natural and artificial sources. Natural background radiation (NBR) may come from radon and its daughter products, crust, cosmic rays, soil, water, food etc. According to the United States report in 2008, the average world population exposure to natural background radiation was about 2.4 millisievert (mSv) per annum, whereas additional radiation contributed from medical diagnosis is estimated at about 0.6 mSv annually (UNSCEAR, 2010). Radiation injuries, such as epithelial and stromal lesion, vascular lesions, fibrosis, and neoplasia may occur upon irradiation (Fajardo, 2005), and level of injuries mainly depends on the radiation dose, duration, and cell cycle stage. In general, male and female gametes are more susceptible to radiation which may affect the composition and biological integrity of their proteins, lipids, and nucleic acids, and hence compromising their capacity to produce normal embryos (García-Rodríguez et al., 2018). Indeed, the adverse effects of ionizing radiation on the reproductive system of mammals (including human being) have been clearly demonstrated by numerous studies. Radiation, for example, has been shown to reduce testis weight and sperm count in male rodent (Haines et al., 2002). In human, a radiation dose as low as 1 Gy can disrupt gonadotropin levels, reduce the number of spermatocytes and spermatids, while a higher radiation dose of 10 Gy can kill all spermatogonial stem cells, leading to permanent azoospermia (Rowley et al., 1974; Clifton and Bremner, 1983). *In vivo* studies in female rats demonstrated that radiation can reduce a number of germ cells and increase synaptonemal complex (SC) fragmentation (Pujol et al., 1996, 1997). Female cancer patients after irradiation treatments often experience premature ovarian failure, infertility, uterine damage, premature deliveries, and miscarriage (Critchley et al., 1992; Wallace et al., 2003).

At cellular level, ionizing radiation can induce DNA damage: (i) directly, *via* energy deposition to DNA molecules; and (ii) indirectly, *via* the attack from reactive oxygen species generated by other ionized molecules, such as free radicals (Ward, 1988). DNA breaks, if failed to repair, can increase the risk of mutagenesis and carcinogenesis (Goodhead, 1994; Ward, 1995; Little, 2000). Haploid germ cells are more susceptible to DNA mutation induced by radiation due to the absence of sister chromatid. DNA repair is thus performed by non-homologous end joining, which is generally accepted as an error-prone pathway (Ahmed et al., 2015; Wdowiak et al., 2019).

Apart from DNA damage, emerging evidence showed that ionizing radiation may further alter DNA methylation pattern (Miousse et al., 2017), modify histone and chromatin structure (Rogakou et al., 1998; Pogribny et al., 2005; Kumar R. et al., 2013), and miRNA expression levels (Halimi et al., 2012; Metheetrairut and Slack, 2013). These induced changes in paternal epigenome can be passed on and affect fertilization and embryogenesis in subsequent generations (Jenkins and Carrell, 2012), thereby causing transgenerational effects. Non-coding RNA is suggested to play a role in epigenetic inheritance by the trafficking of microRNA-containing vesicles to sperm *via* blood stream (Paris et al., 2015; Grandjean et al., 2016; Sharma et al., 2018; Szatmári et al., 2019). In zebrafish, it has been shown that differential methylation of specific genes in F0 caused by parental radiation exposure can be transmitted to their F3 offspring, which had never been exposed to radiation before throughout their life cycle (Kamstra et al., 2018). Nevertheless, existing knowledge on radiation-induced epigenetics alterations on reproductive system, especially female, are limited. Irradiated (2.5 Gy) mice showed an upregulation of miR-29 family in male germline, resulting in reduced expression of *de novo* DNA methyltransferase 3a and hypomethylation of interspersed nuclear elements associated to chromatin modification (Filkowski et al., 2010). Increased phosphorylation of histone H2AX (γ-H2AX) was observed in immature spermatozoa of cranially exposed (20 Gy) rats *via* bystander effect. Significant reduction in global DNA methylation was also found in testes and mature sperm cells (Tamminga et al., 2008). On the contrary, significant increase in global methylation was found in spermatozoa of a human exposed to occupational radiation (Kumar D. et al., 2013). Nevertheless, the epigenetic and transgenerational effects induced by radiation on the reproductive system are still not fully understood, and the underlying mechanisms remained obscure and need further studies (Skrzypek et al., 2019; Dubrova and Sarapultseva, 2020).

Using two different types of human ovarian cells and two different types of testicular cells as models, we carried out a series of *in vitro* experiments followed by bioinformatics analysis (including gene ontology functional analysis and Ingenuity Pathway Analysis), to unravel and compare epigenetic effects and pathway changes in male and female reproductive cells caused by environmentally relevant dose of ionizing radiation. Specifically, we hypothesize that: (a) ionizing radiation could alter the expression of gene cluster related to DNA damage response, and (b) reproductive impairment caused by ionizing radiation is gender specific and controlled by different gene networks.

## Materials and Methods

### Ovarian and Testicular Cell Culture and Ionizing Radiation Exposure

Two human ovarian cancer cells (SKOV3 and COV434) and two mouse testicular germ cells (GC-1 and TM4) were cultured under the conditions described in Supplementary Table 1. Only human and mouse cell lines (not primary cells) were used, and these were purchased from an international company. In accordance with the national legislation and the institutional requirements, the Human Research Ethics Committee of The University of Hong Kong waived the requirement for ethical approval and written informed consent for participants in this study. The cells were cultured at 37°C under 95% air and 5% carbon dioxide. For the ionizing radiation exposure, the cells were seeded onto six-well plate 1 day before exposure to 10 cGy of X-ray (320 kV, 2 mA) for 1 min (X-RAD 320 X-ray system).

### Cell Viability Test

Cells were seeded in a 96-well plate (24 replicate wells for each treatment). After ionizing radiation exposure, cell viability was measured by the MTT assay (Sigma). Colorimetric reaction was measured at 570 nm.

### RNA Isolation

After radiation exposure, total RNA of the cells was extracted using TRIzol Reagent (Invitrogen) according to the manufacturer’s instructions. Briefly, the cells were lysed in 1 ml of TRIzol. Then, 200 μl of chloroform was added, and the sample was centrifuged at 12,000 *× g* for 15 min. Next, 500 μl of the aqueous phase was mixed with 500 μl of isopropanol and stored in −20°C. After overnight precipitation, the mixture was centrifuged at 12,000 × *g* for 20 min. The RNA pellet was then washed twice using ice-cold 70% ethanol, followed by resuspension in RNAse-free distilled water. RNA quality was assessed using an Agilent 2100 Bioanalyzer system, and samples with an RNA integrity number (RIN) greater than eight was used for RNA library construction.

### RNA Sequencing and Bioinformatics Analysis

RNA (cDNA) libraries (three biological replicates from each treatment) of cells were constructed as previously described (Li et al., 2021) and sequenced by the Beijing Genomics Institute (Wuhan, China). Single-end 50-bp read-length reads were sequenced on a BGISEQ-500RS sequencer. Sequence reads were dynamically trimmed according to the q algorithm of BWA (Li et al., 2021). At least 50 million quality-trimmed reads were obtained in each sample. Quality-trimmed sequence reads were mapped to human genome reference (GRCh38/hg38) for SKOV3 and COV434 cell lines, and mouse genome reference (GRCm39/mm39) for GC-1 and TM4 cell lines. Read-count data was then subjected to differential expression analysis using the edgeR package (Robinson et al., 2010). Genes with FDR < 0.05 were considered differentially expressed genes (DEGs). Furthermore, Gene Ontology (GO) enrichment analysis, Kyoto Encyclopedia of Genes and Genomes (KEGG) pathway analysis, and Ingenuity Pathway Analysis (IPA^®^, QIAGEN^1^) were used to decipher the biological effects and possible epigenetic effect of ionizing radiation on the female and male reproductive systems.

### Data Availability

Sequencing data of transcriptome sequencing that support the findings of this study have been deposited in the NCBI BioProject database^2^ with the BioProject accession codes PRJNA730377.

### Statistical Analysis

For bioinformatics analysis, all pathways, diseases, or biofunctions with *p* < 0.05 was considered statistically significant. In MTT assay, results were compared using paired Student’s *t*-test. All statistical analyses were performed using GraphPad Prism 3.02 (GraphPad Software Inc.), and results with *p* < 0.05 was considered statistically significant.

## Results

### Environmental Relevant Dose of Ionizing Radiation Has no Effect on the Viability of the Female and Male Reproductive Cells

**FIGURE 1:**
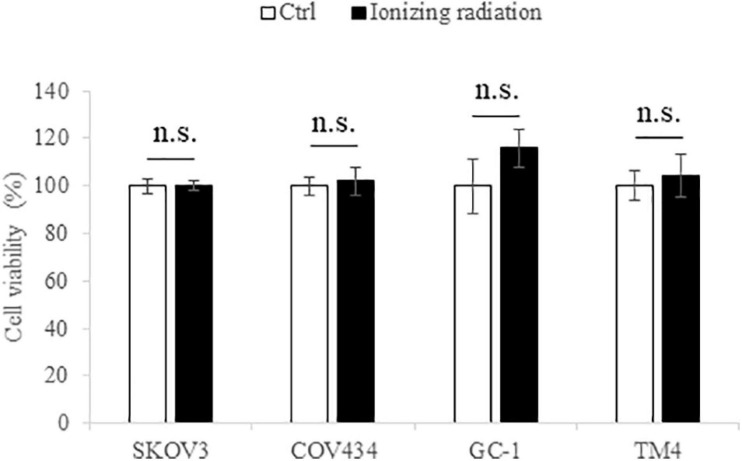
The ionizing radiation exposure (10 cGy) has no effect on the cell viability of SKOV3, COV434, GC-1, and TM4 cells. The white bar represented the control group; the black bar represented the ionizing radiation group. N.s., not statistically significant.

MTT assay was employed to determine the cytotoxicity of the tested ionizing radiation (10 cGy). No significant change in cell viability was found after ionizing radiation exposure (Figure 1), suggesting that the level of radiation used in this study had no cytotoxic effect on both female and male reproductive cells.

### Ionizing Radiation Induced Differential Gene Expression in Both Female and Male Reproductive Cells

**FIGURE 2:**
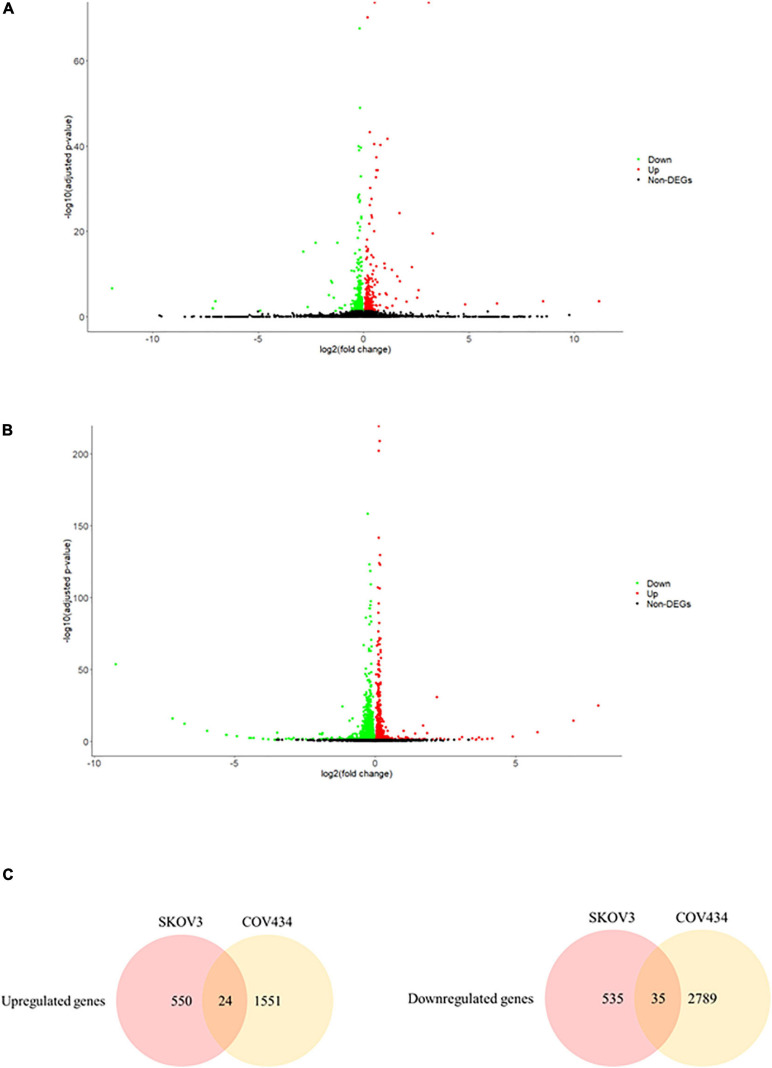
The ionizing radiation exposure caused the differential gene expression in male reproductive cells. **(A)** Volcano plot showed the differentially expressed genes in the TM4 cells. Green dots represented downregulated genes. Red dots represented upregulated genes. **(B)** Volcano plot showed the differentially expressed genes in the GC-1 cells. Green dots represented downregulated genes. Red dots represented upregulated genes. **(C)** Common deregulated genes in TM4 and GC-1 cells under the ionizing radiation exposure.

**FIGURE 3:**
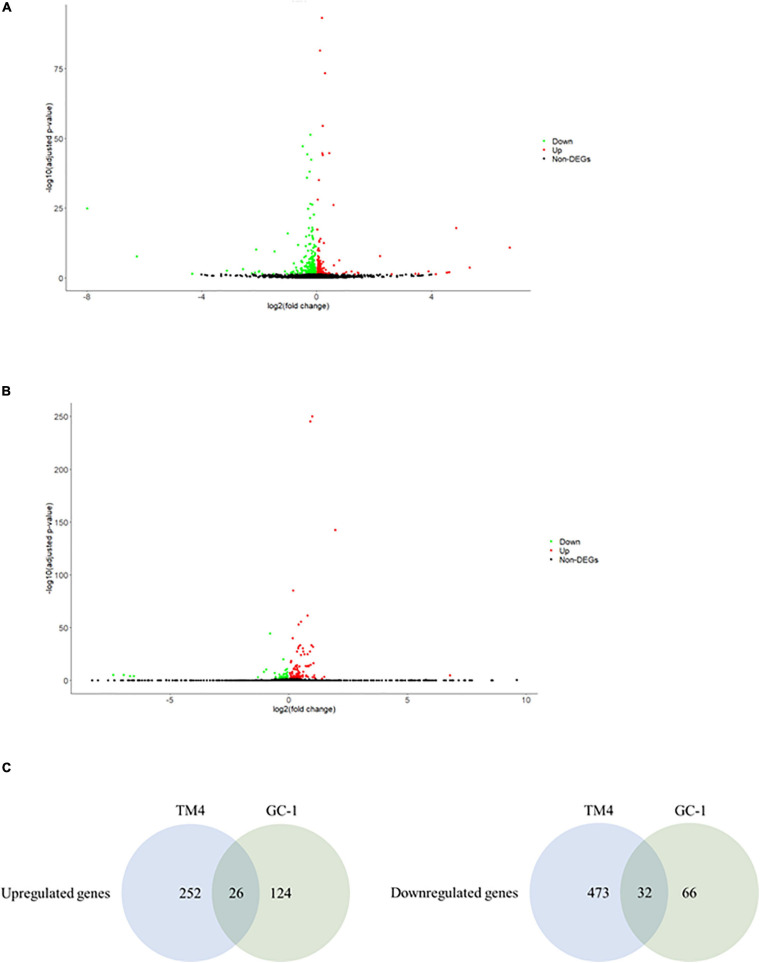
The ionizing radiation exposure caused the differential gene expression in female reproductive cells. **(A)** Volcano plot showed the differentially expressed genes in the SKOV3 cells. Green dots represented downregulated genes. Red dots represented upregulated genes. **(B)** Volcano plot showed the differential expressed genes in the COV434 cells. Green dots represented downregulated genes. Red dots represented upregulated genes. **(C)** Common deregulated genes in SKOV3 and COV434 cells under the ionizing radiation exposure.

In an attempt to understand the biological functions altered by the ionizing radiation, a comparative transcriptomic analysis was conducted. Deep sequencing of RNA libraries derived from control and ionizing radiation treatment groups of each cell lines generated at least 42 million quality-trimmed clean reads (Supplementary Table 2). A total of 51.2 Gb quality-trimmed bases were obtained from the transcriptome sequencing (Supplementary Table 2). Over 95% of sequencing reads could be mapped to the reference genome (Supplementary Table 2). Comparative transcriptomic analysis of ovarian cancer SKOV3 cells found a total 1,144 differentially expressed genes (DEGs), including 574 upregulated and 570 downregulated genes in the ionizing radiation group as compared with the control group (Figure 2A and Supplementary Table 3). Four thousand three hundred ninety-nine DEGs, including 1,575 upregulated and 2,824 downregulated genes were found in ovarian cancer COV434 cells after ionizing radiation exposure (Figure 2B and Supplementary Table 4). Upregulation of 24 genes and downregulation of 35 genes were common in SKOV3 and COV434 after ionizing radiation (Figure 2C and Table 1). Comparative transcriptomic analysis further revealed a smaller number of DEGs in male reproductive cell lines compared with female ovarian cancer cell lines after ionizing radiation. In testicular TM4 germ cell, a total of 783 DEGs, including 278 upregulated and 505 downregulated genes, were found in ionizing radiation group as compared with control group (Figure 3A and Supplementary Table 5). In another testicular GC-1 germ cell, we found 248 DEGs including 150 upregulated and 98 downregulated genes under ionizing radiation (Figure 3B and Supplementary Table 6). Notably, TM4 and GC-1 cells shared 26 upregulated and 32 downregulated genes (Figure 3C and Table 2).

**TABLE 1:** Common deregulated genes in SKOV3 and COV434 after radiation exposure.

Common	
ABHD14A-ACY1	6.321928095
AGAP6	−0.26995382
AHNAK	−0.036692267
ANKRD52	−0.125579585
BHLHE40	0.159297821
CCNB1	0.118534794
CCT8	0.112181258
CELSR1	−0.169768603
CEP170B	−0.203081668
CLK1	−0.581517732
CYR61	0.50372215
DAG1	−0.137058218
DNAJB1	0.421062267
FLNA	−0.137776264
FLNB	−0.101035897
HCFC1	−0.256265359
HMGA2	−0.279167524
HSPA1A	0.674635288
HSPA1B	0.802124194
HSPA8	0.522838677
HSPE1-MOB4	1.696165063
HSPG2	−0.131589484
ILF3	−0.117865207
KMT2D	−0.128633535
LARP1	−0.130260559
LDHA	0.070238521
LENG8	−0.167621054
MAP3K14	−0.246671519
MSH5	−1.036220188
MTRNR2L1	0.086443006
MTRNR2L2	0.055259141
MTRNR2L6	0.366614351
MTRNR2L8	0.07506049
MYC	0.465690304
NACA	0.066625449
NBPF11	−2.273922722
NDST1	−0.324568416
NOL9	−0.227755207
NOMO3	0.261103909
PKD1	−0.212287115
PLEC	−0.211283151
POLE	−0.149276115
PPP1R15A	0.341577734
PRRC2B	−0.102852978
RHOB	0.332811579
RPS17	8.515699838
SH3BP4	−0.306432533
SMAD3	−0.169052451
SNRNP200	−0.082025905
SRCAP	−0.18936661
SRRM2	−0.08339425
TCP1	0.131439798
TJP1	−0.138449491
TMEM189-UBE2V1	−4.906890596
TNRC18	−0.247618398
TPT1	0.082415032
TPX2	0.109746859
TRIO	−0.102527386
UBR4	−0.100565996

**TABLE 2:** Common deregulated genes in TM4 and GC-1 after radiation exposure.

Common	Genes
6820431F20Rik	−0.148053365
Actb	0.015429673
Actg1	0.035451843
Amotl2	0.117501459
Atad5	−0.144339873
Atrx	−0.155713413
Bclaf1	−0.091555479
Cd209c	−0.538806202
Cd2ap	−0.108090558
Cited2	0.074274825
Cmah	−0.055197597
Col1a1	0.115874807
Ctgf	0.213118333
Cxcr2	0.239034728
Cyr61	0.184896033
Ddx17	−0.121821168
Dst	−0.118465395
Dusp1	0.227403063
Egr1	0.294032038
Eif1	0.081156225
Eif4a2	−0.147533967
Flnb	0.044000894
Fn1	0.03142685
Fubp1	−0.054877449
Hspa8	0.027844383
Ier2	0.169029369
Ier3	0.226920302
Luc7l3	−0.306875903
Mat2a	−0.098084338
Mki67	−0.187693922
Myc	0.09462868
Ncl	−0.055239947
Npm1	−0.02494927
Pcm1	−0.107611547
Phlda1	0.066301904
Plk2	0.129986464
Psip1	−0.107652929
Ptgs2	0.085702
Ranbp2	−0.106473682
Rhob	0.100308303
Rplp0	0.014678559
Scaf11	−0.053057537
Serpine1	0.060359909
Setd2	−0.085843794
Smarca5	−0.063291942
Smc2	−0.18366615
Smc3	−0.112639541
Smc4	−0.145773918
Sparc	0.081296273
Srsf10	−0.063547185
Thbs1	0.048740214
Tmem254c	−7.996389141
Top2a	−0.085094243
Trpm7	−0.156997671
Ttc14	−0.263145409
U2surp	−0.094367453
Ubc	0.050980317
Zfc3h1	−0.170572408

### Ionizing Radiation Altered Biological Processes and Signaling Pathways in the Reproductive Cells

**FIGURE 4:**
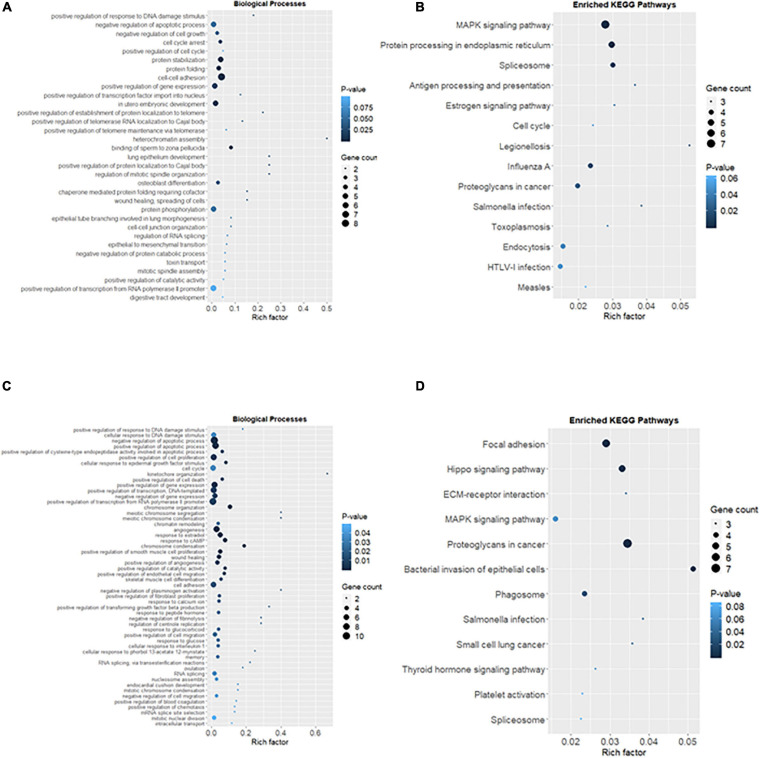
Alteration of biological processes and signaling pathways in the reproductive cells caused by the ionizing radiation. **(A)** Rich factor plot showed the altered biological processes related to DNA damage responses and cell apoptosis and telomere structure in female reproductive cells using Gene Ontology (GO) enrichment analysis. **(B)** Rich factor plot showed the altered cell signaling pathways in the female reproductive cells after the ionizing radiation exposure using Kyoto Encyclopedia of Genes and Genomes (KEGG) analysis. **(C)** Rich factor plot showed the altered biological processes related to DNA damage and chromosome organization in male reproductive cells using GO enrichment analysis. **(D)** Rich factor plot showed the altered cell signaling pathways in the male reproductive cells after the ionizing radiation exposure using KEGG analysis. The size of dot represented the number of gene. The color intensity of dot represented the significance of the biological processes and cell signaling pathways.

The common DEGs were then subjected to GO and KEGG enrichment analysis, to elucidate the alteration of biological functions and signaling pathways in female and male reproductive cells caused by the ionizing radiation. The result of GO analysis in the female reproductive cells showed that the ionizing radiation would trigger DNA damage response, leading to the alteration of many biological processes related to cell apoptosis, cell growth, cell cycle arrest, protein stabilization and folding, cell-cell adhesion, gene expression, and *in utero* embryonic development (Figure 4A and Table 3). More importantly, our results showed that ionizing radiation can alter the biological processes closely related to epigenetic regulation such as establishment of protein localization to telomere, telomerase RNA localization to Cajal body, and heterochromatin assembly (Figure 4A and Table 3). Results of KEGG pathway analysis further highlighted that radiation causes alteration of MAPK signaling pathway, protein processing in endoplasmic reticulum, spliceosome, antigen processing and presentation, estrogen signaling pathway, and cell cycle (Figure 4B and Table 4). In the male reproductive cells, the ionizing radiation could similarly cause a DNA damage response (Figure 4C and Table 5), leading to alterations of different biological processes such as cell apoptosis, cell proliferation, cell cycle, and cell death (Figure 4C and Table 5). Although the responses were similar to those observed in female reproductive cells, the gene clusters involved in the processes were largely different (Table 6) and only shared the induction of MYC and CYR61 (Table 6). More importantly, the ionizing radiation resulted in the modulation of chromatin remodeling such as chromosome organization, meiotic chromosome segregation, and meiotic chromosome condensation, which was considered a major event leading to epigenetic modification in male reproductive cells (Figure 4C and Table 5). The chromatin remodeling is controlled by a family of structural maintenance of chromosome protein including SMC2, SMC3, and SMC4 and SJQ/SMB-related matrix-associated actin-dependent regulator of chromatin subfamily A member 5 (SMARCA5) (Table 5). The result of KEGG pathway analysis highlighted the alteration of focal adhesion, Hippo signaling pathway, ECM-receptor interaction, and MAPK signaling pathway (Figure 4D and Table 7). Taken together, our data suggested that ionizing radiation caused a similar biological alteration in both the female and male reproductive cells through the regulation of different gene clusters.

**TABLE 3:** Gene ontology enrichment analysis on SKOV3 and COV434 common deregulated genes.

Term	Count	*P*-value	Genes
GO:2001022∼positive regulation of response to DNA damage stimulus	2	0.026451	MYC, HMGA2
GO:0043066∼negative regulation of apoptotic process	5	0.048141	SMAD3, FLNA, HMGA2, CYR61, HSPA1B
GO:0030308∼negative regulation of cell growth	3	0.037132	SMAD3, SH3BP4, HSPA1A
GO:0007050∼cell cycle arrest	3	0.016601	SMAD3, MYC, PKD1
GO:0045787∼positive regulation of cell cycle	2	0.092951	CCNB1, MYC
GO:0050821∼protein stabilization	5	2.61E-04	SMAD3, TCP1, FLNA, CCT8, HCFC1
GO:0006457∼protein folding	4	0.003713	DNAJB1, HSPA8, TCP1, CCT8
GO:0098609∼cell–cell adhesion	8	3.37E-07	TJP1, DNAJB1, LDHA, LARP1, FLNB, HCFC1, PLEC, HSPA1A
GO:0010628∼positive regulation of gene expression	5	0.015824	PPP1R15A, HSPA8, SMAD3, HMGA2, HCFC1
GO:0042993∼positive regulation of transcription factor import into nucleus	2	0.038247	SMAD3, FLNA
GO:0001701∼*in utero* embryonic development	5	0.005915	KMT2D, CCNB1, SMAD3, MYC, PKD1
GO:1904851∼positive regulation of establishment of protein localization to telomere	2	0.021693	TCP1, CCT8
GO:1904874∼positive regulation of telomerase RNA localization to Cajal body	2	0.035899	TCP1, CCT8
GO:0032212∼positive regulation of telomere maintenance via telomerase	2	0.075063	TCP1, CCT8
GO:0031507∼heterochromatin assembly	2	0.009699	HMGA2, TNRC18
GO:0007339∼binding of sperm to zona pellucida	3	0.003459	TCP1, CCT8, HSPA1B
GO:0060428∼lung epithelium development	2	0.019306	HMGA2, PKD1
GO:1904871(positive regulation of protein localization to Cajal body	2	0.019306	TCP1, CCT8
GO:0060236(regulation of mitotic spindle organization	2	0.019306	TPX2, PKD1
GO:0001649(osteoblast differentiation	3	0.030878	SMAD3, SNRNP200, CYR61
GO:0051085(chaperone mediated protein folding requiring cofactor	2	0.031186	DNAJB1, HSPA8
GO:0044319(wound healing, spreading of cells	2	0.031186	FLNA, CYR61
GO:0006468(protein phosphorylation	5	0.050757	CLK1, ILF3, CCNB1, TRIO, MAP3K14
GO:0060441(epithelial tube branching involved in lung morphogenesis	2	0.05683	DAG1, HMGA2
GO:0045216(cell—cell junction organization	2	0.05683	SMAD3, FLNA
GO:0043484∼regulation of RNA splicing	2	0.068266	CLK1, AHNAK
GO:0001837∼epithelial to mesenchymal transition	2	0.072803	FLNA, HMGA2
GO:0042177∼negative regulation of protein catabolic process	2	0.081811	SMAD3, FLNA
GO:1901998∼toxin transport	2	0.081811	TCP1, CCT8
GO:0090307∼mitotic spindle assembly	2	0.081811	TPX2, FLNA
GO:0043085∼positive regulation of catalytic activity	2	0.090733	HSPA8, MYC
GO:0045944∼positive regulation of transcription from RNA polymerase II promoter	6	0.092546	KMT2D, SMAD3, MYC, HMGA2, PKD1, CYR61
GO:0048565∼digestive tract development	2	0.097369	CCNB1, PKD1

**TABLE 4:** KEGG enrichment analysis on SKOV3 and COV434 common deregulated genes.

Term	Count	*P*-value	Genes
mmu04010:MAPK signaling pathway	7	7.20E-05	HSPA8, MYC, FLNA, FLNB, MAP3K14, HSPA1B, HSPA1A
mmu04141:protein processing in endoplasmic reticulum	5	0.001406	PPP1R15A, DNAJB1, HSPA8, HSPA1B, HSPA1A
mmu03040:spliceosome	4	0.006952	HSPA8, SNRNP200, HSPA1B, HSPA1A
mmu04612:antigen processing and presentation	3	0.024577	HSPA8, HSPA1B, HSPA1A
mmu04915:estrogen signaling pathway	3	0.034176	HSPA8, HSPA1B, HSPA1A
mmu04110:cell cycle	3	0.052334	CCNB1, SMAD3, MYC
mmu05134:legionellosis	3	0.012357	HSPA8, HSPA1B, HSPA1A
mmu05164:influenza A	4	0.013793	DNAJB1, HSPA8, HSPA1B, HSPA1A
mmu05205:proteoglycans in cancer	4	0.021748	MYC, FLNA, FLNB, HSPG2
mmu05132:*Salmonella* infection	3	0.022385	TJP1, FLNA, FLNB
mmu05145:toxoplasmosis	3	0.038769	HSPA8, HSPA1B, HSPA1A
mmu04144:endocytosis	4	0.04145	HSPA8, SMAD3, HSPA1B, HSPA1A
mmu05166:HTLV-I infection	4	0.047645	SMAD3, MYC, MAP3K14, POLE
mmu05162:measles	3	0.06166	HSPA8, HSPA1B, HSPA1A

**TABLE 5:** Gene ontology enrichment analysis on GC-1 and TM4 common deregulated genes.

Term	Count	*p*-value	Genes
GO:2001022∼positive regulation of response to DNA damage stimulus	2	0.031782	BCLAF1, MYC
GO:0006974∼cellular response to DNA damage stimulus	5	0.03441	TOP2A, ATAD5, MYC, ATRX, SMC3
GO:0043066∼negative regulation of apoptotic process	10	3.51E-05	NPM1, CITED2, DUSP1, NCL, PLK2, CXCR2, FN1, THBS1, CYR61, IER3
GO:0043065∼positive regulation of apoptotic process	8	5.22E-05	TOP2A, BCLAF1, DUSP1, TRPM7, PTGS2, PHLDA1, CYR61, RHOB
GO:0043280∼positive regulation of cysteine-type endopeptidase activity involved in apoptotic process	3	0.008723	MYC, CYR61, CTGF
GO:0008284∼positive regulation of cell proliferation	7	0.004907	NPM1, MYC, CXCR2, FN1, PTGS2, THBS1, CTGF
GO:0071364∼cellular response to epidermal growth factor stimulus	3	0.004982	COL1A1, NCL, MYC
GO:0007049∼cell cycle	6	0.033428	DUSP1, MKI67, SMC3, SMC4, SMC2, CD2AP
GO:0051383∼kinetochore organization	2	0.008768	SMC4, SMC2
GO:0010942∼positive regulation of cell death	3	0.008723	EGR1, PTGS2, CTGF
GO:0010628∼positive regulation of gene expression	7	0.001063	EGR1, HSPA8, CITED2, FUBP1, SERPINE1, FN1, CTGF
GO:0045893∼positive regulation of transcription, DNA-templated	7	0.006565	DDX17, COL1A1, EGR1, NPM1, CITED2, MYC, SMARCA5
GO:0010629∼negative regulation of gene expression	5	0.007523	NPM1, CITED2, MYC, SERPINE1, CTGF
GO:0045944∼positive regulation of transcription from RNA polymerase II promoter	9	0.007997	DDX17, TOP2A, EGR1, CITED2, NCL, MYC, ATRX, PSIP1, CYR61
GO:0051276∼chromosome organization	4	1.87E-04	MYC, SMC3, SMC4, SMC2
GO:0045132∼meiotic chromosome segregation	2	0.014571	SMC4, SMC2
GO:0010032∼meiotic chromosome condensation	2	0.014571	SMC4, SMC2
GO:0006338∼chromatin remodeling	3	0.022492	MYC, ATRX, SMARCA5
GO:0001525∼angiogenesis	7	6.84E-05	SETD2, NCL, SERPINE1, FN1, PTGS2, CTGF, RHOB
GO:0032355∼response to estradiol	5	2.18E-04	COL1A1, DUSP1, MYC, PTGS2, CTGF
GO:0051591∼response to cAMP	4	4.48E-04	COL1A1, SPARC, MAT2A, DUSP1
GO:0030261∼chromosome condensation	3	9.85E-04	TOP2A, SMC4, SMC2
GO:0048661∼positive regulation of smooth muscle cell proliferation	4	0.001666	EGR1, MYC, PTGS2, THBS1
GO:0042060∼wound healing	4	0.002641	COL1A1, SPARC, SERPINE1, FN1
GO:0045766∼positive regulation of angiogenesis	4	0.005367	SERPINE1, CXCR2, THBS1, RHOB
GO:0043085∼positive regulation of catalytic activity	3	0.005828	HSPA8, NPM1, MYC
GO:0010595∼positive regulation of endothelial cell migration	3	0.006425	SPARC, THBS1, RHOB
GO:0035914∼skeletal muscle cell differentiation	3	0.011335	EGR1, CITED2, MYC
GO:0007155∼cell adhesion	6	0.013561	DST, FN1, THBS1, CYR61, CTGF, RHOB
GO:0010757∼negative regulation of plasminogen activation	2	0.014571	SERPINE1, THBS1
GO:0048146∼positive regulation of fibroblast proliferation	3	0.015584	MYC, SERPINE1, FN1
GO:0051592∼response to calcium ion	3	0.016973	SPARC, DUSP1, THBS1
GO:0071636∼positive regulation of transforming growth factor beta production	2	0.017461	PTGS2, THBS1
GO:0043434∼response to peptide hormone	3	0.0194	COL1A1, SPARC, CTGF
GO:0051918∼negative regulation of fibrinolysis	2	0.020341	SERPINE1, THBS1
GO:0046599∼regulation of centriole replication	2	0.020341	NPM1, PLK2
GO:0051384∼response to glucocorticoid	3	0.020409	SPARC, DUSP1, PTGS2
GO:0030335∼positive regulation of cell migration	4	0.021653	COL1A1, FN1, THBS1, CYR61
GO:0009749∼response to glucose	3	0.021963	EGR1, THBS1, CTGF
GO:0071347∼cellular response to interleukin-1	3	0.023026	MYC, SERPINE1, FN1
GO:1904628∼cellular response to phorbol 13-acetate 12-myristate	2	0.023214	MYC, RPLP0
GO:0007613∼memory	3	0.024659	PLK2, TRPM7, PTGS2
GO:0000375∼RNA splicing, via transesterification reactions	2	0.026078	SCAF11, SRSF10
GO:0030728∼ovulation	2	0.031782	MYC, PTGS2
GO:0008380∼RNA splicing	4	0.033622	HSPA8, SCAF11, LUC7L3, SRSF10
GO:0006334(nucleosome assembly	3	0.037334	NPM1, ATRX, SMARCA5
GO:0003197(endocardial cushion development	2	0.037453	CITED2, THBS1
GO:0007076(mitotic chromosome condensation	2	0.037453	SMC4, SMC2
GO:0030336(negative regulation of cell migration	3	0.038648	CITED2, SERPINE1, RHOB
GO:0030194(positive regulation of blood coagulation	2	0.040277	SERPINE1, THBS1
GO:0050921(positive regulation of chemotaxis	2	0.043092	FN1, THBS1
GO:0006376(mRNA splice site selection	2	0.043092	LUC7L3, SRSF10
GO:0007067(mitotic nuclear division	4	0.047549	SMC3, SMC4, SMC2, CD2AP
GO:0046907(intracellular transport	2	0.048698	RANBP2, DST
GO:0070542(response to fatty acid	2	0.054272	PTGS2, CTGF
GO:0006259(DNA metabolic process	2	0.057047	TOP2A, MKI67
GO:0046697(decidualization	2	0.068067	CITED2, PTGS2
GO:0007126(meiotic nuclear division	2	0.068067	MKI67, SMC3
GO:0030511(positive regulation of transforming growth factor beta receptor signaling pathway	2	0.068067	CITED2, THBS1
GO:0043044(ATP-dependent chromatin remodeling	2	0.070802	SMARCA5, ACTB
GO:0001895(retina homeostasis	2	0.070802	ACTB, ACTG1
GO:0048025(negative regulation of mRNA splicing, via spliceosome	2	0.070802	NPM1, SRSF10
GO:0010667∼negative regulation of cardiac muscle cell apoptotic process	2	0.07353	HSPA8, NPM1
GO:0016925∼protein sumoylation	2	0.076249	TOP2A, RANBP2
GO:0042493∼response to drug	4	0.077109	COL1A1, MAT2A, PTGS2, THBS1
GO:0007052∼mitotic spindle organization	2	0.078961	PLK2, SMC3
GO:0007569∼cell aging	2	0.084361	NPM1, CITED2
GO:0001937∼negative regulation of endothelial cell proliferation	2	0.084361	SPARC, THBS1
GO:0044344∼cellular response to fibroblast growth factor stimulus	2	0.084361	COL1A1, MYC
GO:0070372∼regulation of ERK1 and ERK2 cascade	2	0.087049	FN1, CYR61
GO:0033574∼response to testosterone	2	0.089729	DUSP1, THBS1
GO:0006446∼regulation of translational initiation	2	0.092402	EIF4A2, EIF1
GO:0006351∼transcription, DNA-templated	10	0.095659	DDX17, EGR1, HSPA8, BCLAF1, SETD2, CITED2, MYC, FUBP1, ATRX, PSIP1
GO:0051301∼cell division	4	0.09664	SMC3, SMC4, SMC2, CD2AP

**TABLE 6:** Different gene clusters involved in similar biological processes.

SKOV3/COV434	Biological processes	TM4/GC-1
MYC, HMGA2	DNA damage response	BCLAF1, TOP2A, ATAD5, MYC, ATRX, SMC3
SMAD3, FLNA, HMGA2, CYR61, HSPA1B	Cell apoptosis	NPM1, CITED2, DUSP1, NCL, PLK2, CXCR2, FN1, THBS1, CYR61, IER3, MYC, CTGF, TOP2A, BCLAF1, TRPM7, PTGS2, PHLDA1, RHOB
SMAD3, MYC, PKD1, CCNB1	Cell cycle	DUSP1, MKI67, SMC3, SMC4, SMC2, CD2AP, MYC
SMAD3, SH3BP4, HSPA1A	Cell proliferation	NPM1, MYC, CXCR2, FN1, PTGS2, THBS1, CTGF, COL1A1, NCL,
PPP1R15A, HSPA8, SMAD3, HMGA2, HCFC1, FLNA	Gene expression	EGR1, HSPA8, CITED2, FUBP1, SERPINE1, FN1, CTGF, NPM1, MYC, DDX17, COL1A1, SMARCA5

**TABLE 7:** KEGG enrichment analysis on GC-1 and TM4 common deregulated genes.

Term	Count	*p*-value	Genes
mmu04510:focal adhesion	6	7.98E-04	COL1A1, FN1, FLNB, THBS1, ACTB, ACTG1
mmu04390:hippo signaling pathway	5	0.002025	MYC, SERPINE1, ACTB, ACTG1, CTGF
mmu04512:ECM-receptor interaction	3	0.040363	COL1A1, FN1, THBS1
mmu04010:MAPK signaling pathway	4	0.061762	HSPA8, DUSP1, MYC, FLNB
mmu05205:proteoglycans in cancer	7	7.28E-05	COL1A1, MYC, FN1, FLNB, THBS1, ACTB, ACTG1
mmu05100:bacterial invasion of epithelial cells	4	0.002739	FN1, ACTB, CD2AP, ACTG1
mmu04145:phagosome	4	0.023537	CD209C, THBS1, ACTB, ACTG1
mmu05132:Salmonella infection	3	0.032375	FLNB, ACTB, ACTG1
mmu05222:small cell lung cancer	3	0.037085	MYC, FN1, PTGS2
mmu04919:thyroid hormone signaling pathway	3	0.064117	MYC, ACTB, ACTG1
mmu04611:platelet activation	3	0.081647	COL1A1, ACTB, ACTG1
mmu03040:spliceosome	3	0.0838	HSPA8, SRSF10, U2SURP

### Ionizing Radiation Caused Deregulation of Gene Network and Reproductive Disorder

**FIGURE 5:**
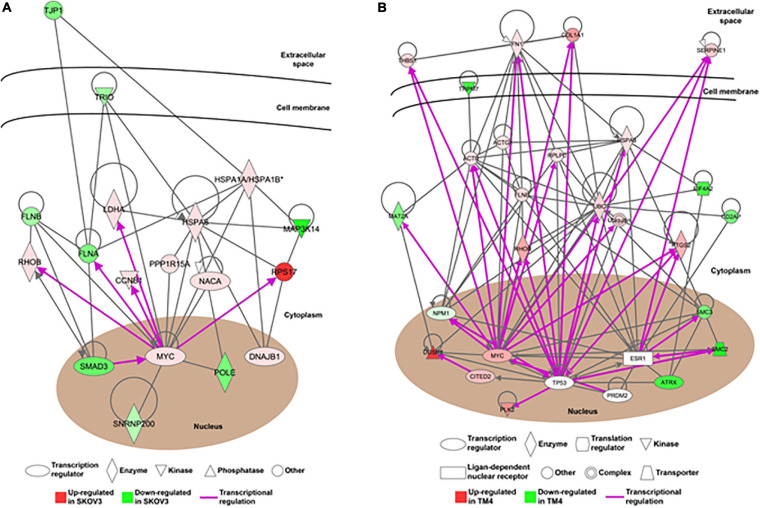
The alteration of gene networks is caused by the ionizing radiation. The gene networking of the Ingenuity Pathway Analysis (IPA) showed the gene networks in response to the ionizing radiation in **(A)** female reproductive cells and **(B)** male reproductive cells. Red color represented upregulated genes and green color represented downregulated genes. The purple arrow showed the direct transcriptional regulation.

In order to understand the reproductive diseases and to delineate the gene networks involved in ionizing radiation-altered biological processes, IPA was conducted. The results of disease analysis showed that the radiation exposure could potentially lead to many female reproductive disorders and diseases such as ovary growth impairment, genital tract cancer, and ovarian cancer (Table 8). A similar finding was observed in the male reproductive system that radiation could cause tumorigenesis of reproductive tract and develop malignant neoplasm and endometriosis of the male genital organ (Table 9). Canonical pathway analysis of IPA further highlighted the involvement of many components from different cellular levels in radiation-mediated reproductive impairment. In the female reproductive cells, tight junction protein 1 (TJP1) was found to be downregulated and associated with the deregulation of filamin A, alpha (FLNA) and heat shock 70 kDa protein 1 (HSPA1A) (Figure 5A). Also, a group of enzymes such as lactate dehydrogenase A (LDHA), DNA polymerase Epsilon (POLE), and small nuclear ribonucleoprotein U5 Subunit 200 (SNRP200) (Figure 5A) were highlighted. More importantly, a cluster of transcription factors including DnaJ heat shock protein family (Hsp40) member B1 (DNAJB1), mothers against decapentaplegic homolog 3 (SMAD3) and MYC proto-oncogene, and BHLH transcription factor (MYC) were involved in the ionizing radiation-mediated reproductive impairment (Figure 5A). Our result showed that MYC is a key mediator that directly controls different kinases and enzymes (Figure 5A). In the male reproductive cells, more candidates were found to be involved in reproductive impairment caused by the ionizing radiation (Figure 5B). The gene networking showed the induction of thrombospondin-1 (THBS1), fibronectin 1 (FN1), collagen, type I, alpha 1 (COL1A1), and serpin family E member 1 (SERPINE1) in extracellular matrix (Figure 5B). Also, radiation exposure could lead to downregulation of membrane protein transient receptor potential cation channel subfamily M member 7 (TRPM7). Similar to the result in the female reproductive cells, MYC was the major mediator connecting different enzymes such as methionine adenosyltransferase 2A (MAT2), Ras homolog family member B (RHOB), ubiquitin C (UBC), prostaglandin-endoperoxide synthase 2 (PTGS2), and heat shock protein family A member 8 (HSPA8), as well as a large number of transcription factors including nucleophosmin (NPM1), Cbp/p300-interacting transactivator 2 (CITED2), and ATP-dependent helicase (ATRX) (Figure 5B). Taken together, our results suggested a gender-specific gene network involved in the ionizing radiation-mediated reproductive impairment.

**TABLE 8:** Female reproductive diseases caused by radiation exposure.

Diseases or functions annotation	*p*-value	# molecules	Molecules
Genital tract cancer	2.64E-08	46	ABHD14A-ACY1, AGAP6 (includes others), AHNAK, ANKRD52, BHLHE40, CCN1, CCNB1, CELSR1, CEP170B, CLK1, FLNA, FLNB, HCFC1, HMGA2, HSPA1A/HSPA1B, HSPA8, HSPE1-MOB4, HSPG2, ILF3, KMT2D, LARP1, LENG8, MAP3K14, MSH5, MYC, NACA, NBPF10 (includes others), NDST1, NOMO1 (includes others), PKD1, PLEC, POLE, PPP1R15A, PRRC2B, RHOB, SH3BP4, SMAD3, SNRNP200, SRCAP, SRRM2, TJP1, TMEM189-UBE2V1, TNRC18, TPX2, TRIO, UBR4
Genital tumor	4.45E-08	46	ABHD14A-ACY1, AGAP6 (includes others), AHNAK, ANKRD52, BHLHE40, CCN1, CCNB1, CELSR1, CEP170B, CLK1, FLNA, FLNB, HCFC1, HMGA2, HSPA1A/HSPA1B, HSPA8, HSPE1-MOB4, HSPG2, ILF3, KMT2D, LARP1, LENG8, MAP3K14, MSH5, MYC, NACA, NBPF10 (includes others), NDST1, NOMO1 (includes others), PKD1, PLEC, POLE, PPP1R15A, PRRC2B, RHOB, SH3BP4, SMAD3, SNRNP200, SRCAP, SRRM2, TJP1, TMEM189-UBE2V1, TNRC18, TPX2, TRIO, UBR4
Female genital tract cancer	1.02E-07	40	ABHD14A-ACY1, AGAP6 (includes others), AHNAK, ANKRD52, CCN1, CCNB1, CELSR1, CLK1, FLNA, FLNB, HCFC1, HMGA2, HSPA1A/HSPA1B, HSPE1-MOB4, HSPG2, ILF3, KMT2D, LARP1, LENG8, MAP3K14, MSH5, MYC, NBPF10 (includes others), NDST1, NOMO1 (includes others), PKD1, PLEC, POLE, RHOB, SH3BP4, SMAD3, SNRNP200, SRCAP, SRRM2, TJP1, TMEM189-UBE2V1, TNRC18, TPX2, TRIO, UBR4
Tumorigenesis of reproductive tract	1.79E-07	40	ABHD14A-ACY1, AGAP6 (includes others), AHNAK, ANKRD52, CCN1, CCNB1, CELSR1, CLK1, FLNA, FLNB, HCFC1, HMGA2, HSPA1A/HSPA1B, HSPE1-MOB4, HSPG2, ILF3, KMT2D, LARP1, LENG8, MAP3K14, MSH5, MYC, NBPF10 (includes others), NDST1, NOMO1 (includes others), PKD1, PLEC, POLE, RHOB, SH3BP4, SMAD3, SNRNP200, SRCAP, SRRM2, TJP1, TMEM189-UBE2V1, TNRC18, TPX2, TRIO, UBR4
Female genital tract adenocarcinoma	2.10E-07	37	ABHD14A-ACY1, AGAP6 (includes others), AHNAK, ANKRD52, CCNB1, CELSR1, CLK1, FLNA, FLNB, HCFC1, HMGA2, HSPA1A/HSPA1B, HSPE1-MOB4, HSPG2, ILF3, KMT2D, LARP1, LENG8, MAP3K14, MSH5, MYC, NBPF10 (includes others), NDST1, NOMO1 (includes others), PKD1, PLEC, POLE, SH3BP4, SMAD3, SNRNP200, SRCAP, SRRM2, TMEM189-UBE2V1, TNRC18, TPX2, TRIO, UBR4
Endometrioid endometrial adenocarcinoma	2.98E-07	33	ABHD14A-ACY1, AGAP6 (includes others), AHNAK, ANKRD52, CELSR1, FLNA, FLNB, HCFC1, HSPA1A/HSPA1B, HSPG2, ILF3, KMT2D, LARP1, LENG8, MAP3K14, MSH5, MYC, NBPF10 (includes others), NDST1, NOMO1 (includes others), PKD1, PLEC, POLE, SH3BP4, SMAD3, SNRNP200, SRCAP, SRRM2, TMEM189-UBE2V1, TNRC18, TPX2, TRIO, UBR4
Endometrial cancer	3.27E-07	35	ABHD14A-ACY1, AGAP6 (includes others), AHNAK, ANKRD52, CCNB1, CELSR1, FLNA, FLNB, HCFC1, HSPA1A/HSPA1B, HSPG2, ILF3, KMT2D, LARP1, LENG8, MAP3K14, MSH5, MYC, NBPF10 (includes others), NDST1, NOMO1 (includes others), PKD1, PLEC, POLE, SH3BP4, SMAD3, SNRNP200, SRCAP, SRRM2, TJP1, TMEM189-UBE2V1, TNRC18, TPX2, TRIO, UBR4
Development of genital tumor	3.67E-07	39	ABHD14A-ACY1, AGAP6 (includes others), AHNAK, ANKRD52, CCN1, CCNB1, CELSR1, CLK1, FLNA, FLNB, HCFC1, HMGA2, HSPA1A/HSPA1B, HSPE1-MOB4, HSPG2, ILF3, KMT2D, LARP1, LENG8, MAP3K14, MSH5, MYC, NBPF10 (includes others), NDST1, NOMO1 (includes others), PKD1, PLEC, POLE, SH3BP4, SMAD3, SNRNP200, SRCAP, SRRM2, TJP1, TMEM189-UBE2V1, TNRC18, TPX2, TRIO, UBR4
Endometrial carcinoma	5.26E-07	34	ABHD14A-ACY1, AGAP6 (includes others), AHNAK, ANKRD52, CELSR1, FLNA, FLNB, HCFC1, HSPA1A/HSPA1B, HSPG2, ILF3, KMT2D, LARP1, LENG8, MAP3K14, MSH5, MYC, NBPF10 (includes others), NDST1, NOMO1 (includes others), PKD1, PLEC, POLE, SH3BP4, SMAD3, SNRNP200, SRCAP, SRRM2, TJP1, TMEM189-UBE2V1, TNRC18, TPX2, TRIO, UBR4
Ovarian tumor	6.44E-05	18	AHNAK, ANKRD52, CCN1, CCNB1, CLK1, HCFC1, HMGA2, HSPE1-MOB4, KMT2D, MYC, PKD1, POLE, RHOB, SMAD3, SNRNP200, SRCAP, TNRC18, TPX2
Ovarian cancer	1.63E-04	17	AHNAK, ANKRD52, CCN1, CCNB1, CLK1, HCFC1, HMGA2, HSPE1-MOB4, KMT2D, MYC, PKD1, POLE, RHOB, SNRNP200, SRCAP, TNRC18, TPX2
Growth of genital organ	4.23E-04	4	KMT2D, MYC, NDST1, SMAD3
Growth of ovary	6.45E-04	3	KMT2D, MYC, SMAD3
Ovarian carcinoma	1.76E-03	14	AHNAK, ANKRD52, CLK1, HCFC1, HMGA2, HSPE1-MOB4, KMT2D, MYC, PKD1, POLE, SNRNP200, SRCAP, TNRC18, TPX2

**TABLE 9:** Male reproductive diseases caused by radiation exposure.

Diseases or functions annotation	*p*-value	# molecules	Molecules
Malignant neoplasm of male genital organ	9.69E-07	30	ATAD5, ATRX, BCLAF1, CD2AP, COL1A1, DDX17, DUSP1, EGR1, FLNB, FN1, HSPA8, IER3, LUC7L3, MKI67, MYC, NPM1, PHLDA1, PLK2, PSIP1, PTGS2, RGPD4 (includes others), SETD2, SMC3, SMC4, SPARC, SRSF10, TOP2A, TRPM7, UBC, ZFC3H1
Endometriosis	3.09E-06	9	ACTB, CITED2, CXCR2, DUSP1, EGR1, FN1, PLK2, PTGS2, TOP2A
Prostate cancer	9.27E-06	28	ATAD5, ATRX, CD2AP, COL1A1, DDX17, DUSP1, EGR1, FLNB, FN1, HSPA8, IER3, LUC7L3, MKI67, MYC, NPM1, PHLDA1, PLK2, PSIP1, PTGS2, RGPD4 (includes others), SETD2, SMC3, SMC4, SPARC, SRSF10, TOP2A, UBC, ZFC3H1
Tumorigenesis of reproductive tract	9.69E-06	36	ACTB, ACTG1, ATAD5, ATRX, BCLAF1, CD2AP, COL1A1, CXCR2, DUSP1, EIF4A2, FLNB, FN1, FUBP1, IER3, LUC7L3, MKI67, MYC, NPM1, PHLDA1, PLK2, PSIP1, PTGS2, RGPD4 (includes others), RHOB, SCAF11, SERPINE1, SETD2, SMARCA5, SMC3, SMC4, THBS1, TOP2A, TRPM7, U2SURP, UBC, ZFC3H1
Genital tumor	1.16E-05	41	ACTB, ACTG1, ATAD5, ATRX, BCLAF1, CD2AP, COL1A1, CXCR2, DDX17, DUSP1, EGR1, EIF4A2, FLNB, FN1, FUBP1, HSPA8, IER3, LUC7L3, MKI67, MYC, NPM1, PHLDA1, PLK2, PSIP1, PTGS2, RGPD4 (includes others), RHOB, SCAF11, SERPINE1, SETD2, SMARCA5, SMC3, SMC4, SPARC, SRSF10, THBS1, TOP2A, TRPM7, U2SURP, UBC, ZFC3H1
Morphology of reproductive system	1.68E-05	11	ATRX, CXCR2, DUSP1, EGR1, FUBP1, MYC, PLK2, PTGS2, SERPINE1, SETD2, THBS1
Prostatic carcinoma	9.71E-05	24	ATAD5, ATRX, CD2AP, COL1A1, DDX17, DUSP1, FLNB, FN1, HSPA8, LUC7L3, MKI67, MYC, NPM1, PHLDA1, PLK2, PTGS2, RGPD4 (includes others), SETD2, SMC3, SMC4, SRSF10, TOP2A, UBC, ZFC3H1
Development of genital tumor	1.75E-04	33	ACTB, ACTG1, ATAD5, ATRX, BCLAF1, CD2AP, COL1A1, CXCR2, EIF4A2, FLNB, FN1, FUBP1, IER3, LUC7L3, MKI67, MYC, NPM1, PHLDA1, PLK2, PSIP1, PTGS2, RGPD4 (includes others), SCAF11, SETD2, SMARCA5, SMC3, SMC4, THBS1, TOP2A, TRPM7, U2SURP, UBC, ZFC3H1

## Discussion

The present study unraveled that epigenetic changes associated with reproductive impairment can be elicited by a low dose of ionizing radiation (i.e., 100 mGy). The dose we used in this study is considered a low-dose radiation because fetal absorbed about 4.9 cGy per procedure during abdominal CT scan, and for pelvis it was as high as 7.9 cGy (Groen et al., 2012). A cohort study of diagnostic medical radiation workers in South Korea showed that the workers absorbed a mean cumulative badge dose of 7.20 mSv (Lee et al., 2021). While the environmental level of ionizing radiation is about 3 mGy/year, high levels of background radiation was commonly reported in some areas around the world (Hendry et al., 2009).

In this study, we determined and compared the effects of radiation on different types of male and female reproductive cells. GC-1 spg is an immortalized type B spermatogonia line and displays characteristics of a stage between type B spermatogonia and primary spermatocytes. TM4 is an immortalized Sertoli cell line and is a somatic cell-type essential for nurturing germ cell development. Due to the low availability of freshly isolated primary ovarian reproductive cells, two ovarian cancer lines derived from ovarian surface epithelium and granulosa cells, respectively, are used. Our comparative transcriptomic analysis revealed that environmental relevant level of ionizing radiation is sufficient to induced DNA damage, leading to cell cycle arrest and cell apoptosis in both female and male reproductive cells. It is not surprising that the genes involved were largely different according to cell types, and only MYC and CYR61 were shared in both female and male reproductive cells. MYC is a transcription factor and contributes to the development of many cancer types (Godwin et al., 2021). In addition, it plays an important role in DNA damage response repair. For instance, MYC formed foci with γ-H2AX to phosphorylate ATM and to mediate DNA-PKcs activity (Cui et al., 2015), leading to enhanced chromosomal and chromatid breaks in response to γ-ray ionizing radiation (Li et al., 2012). In addition, MYC was essential for DNA damage-induced apoptosis through the control of the p53 tumor suppressor protein (Phesse et al., 2014). The other deregulated gene, cysteine-rich angiogenic inducer 61 (CYR61), is an extracellular matrix-associated signaling protein of the CCN intercellular signaling protein family (Lau, 2011). CYR61 is a cell apoptosis and senescence inducer involved in DNA damage response through the regulation of p53 upon genotoxic stress (Feng et al., 2008; Morrison et al., 2009). In addition, CYR61 was a downstream effector of the Hippo signaling pathway that is one the important cell signaling pathways of DNA damage response (Shome et al., 2020). Other than the common DNA damage responses, our result also suggested that the ionizing radiation could cause gene deregulation related to impairment of utero embryonic development in the female reproductive system. Although it has been reported that abdominopelvic ionizing irradiation (>5 Gy) increased the risk of unfavorable neonatal outcomes such as fetal malformation and disturbances of growth or development (Kumar and DeJesus, 2021), our result showed that even a much lower level of ionizing radiation (10 cGy) could also pose adverse effect on embryonic development. In addition, our data highlighted that ionizing radiation could alter estrogen signaling pathway in female reproductive cells. Estrogen signaling pathway is one of the most important pathways involved in steroid hormones and reproductive regulation in mammals (Saito and Cui, 2018), and estrogens are also associated with tumor development, particularly breast and ovary cancers (Ferreira et al., 2009). Studies also demonstrated that estrogen is also related to the DNA repair pathways and DNA integrity (Jiménez-Salazar et al., 2021; Pescatori et al., 2021).

Our results further highlighted that ionized radiation could alter a spectrum of male reproductive function-related cell signaling such as ECM-receptor interaction, Hippo signaling pathway, and MAPK signaling pathway. Extracellular matrix (ECM) receptor interaction pathway has been reported to be associated with spermatogenesis (Gong et al., 2018). In addition, ECM remodeling is required for the testicular development and maturation (Slongo et al., 2002). Hippo signaling pathway is responsible for the controls of organ size through the regulation of cell proliferation and apoptosis (Zhang et al., 2009). Yes-associated protein, the downstream effector of Hippo signaling pathway was reported to modulate the decline of germline stem cells and niche cells (Francis et al., 2019). Also, Hippo signaling cascade was found to be associated with the pubertal development of male reproductive tract and spermatogenesis in sheep (Zhang et al., 2019). MAPK signaling pathway is the key mediator that controls the phosphorylation of many downstream effectors, leading to modulate different cellular functions, including cell proliferation, differentiation, and migration (Joerger-Messerli et al., 2021). Oxidative stress-mediated p38 MAPK signaling pathway was associated with the blood-testis barrier-related junction protein and promoting apoptosis in mice testes (Huang et al., 2021). Moreover, activation of the MAPK signaling pathway was involved in the molecular mechanism of apoptosis in spermatogonia cells (Park et al., 2020). As such, deregulation of these pathways found in the present study suggested different aspects of reproductive impairment caused by the ionizing radiation.

Beside the cell signaling pathways related to reproductive functions, our result also demonstrated that ionizing radiation may cause epigenetic modification in both female and male reproductive cells. In female reproductive cells, telomerase, telomere-binding proteins, and heterochromatin assembly were disturbed by ionizing radiation. In male reproductive cells, chromosome organization such as meiotic chromosome segregation and condensation was interfered. Telomere, a repetitive DNA sequencing at the end of chromosome, protects chromosome from progressive degradation. The length of telomere is controlled by a group of telomere-binding proteins and the enzymatic activity of telomerase. Recent research showed that dysregulated telomerase activation was associated with epigenetic, transcriptional, and posttranscriptional modifications (Dogan and Forsyth, 2021). Lister-Shimauchi et al. (2021) demonstrated that deficiency for telomerase in *Caenorhabditis elegans* resulted in transgenerational shortening of telomeres. In addition, histone modifications including activation markers (H3K4me1 and H3K4me3) and silencing marks (H3K27me3) at distal promoters were telomere length dependent, suggesting that the epigenetic state of telomere-distal promoters could be influenced by telomere length (Mukherjee et al., 2018). Moreover, meiotic chromosome segregation is essential for the maintenance of genomic integrity of gametes and requires functional centromeres that are required for a precise epigenetic inheritance (Mahlke and Nechemia-Arbely, 2020; Tan et al., 2020).

In conclusion, we show that environmental relevant dose of ionizing radiation can alter the expression of gene cluster related to DNA damage response through the control of MYC, which agreed with other studies reporting amplification of MYC gene in somatic cells and cancer cells (Sawey et al., 1987; Mothersill et al., 1991; Käcker et al., 2013; Ginter et al., 2014). The ionizing radiation-mediated reproductive impairment is not only gender specific but also connected with different gene networks and pathways. Due to the limited number of germ cells and their extended period of developmental processes, research on the radiation-induced effects of female germline has been hindered (Skrzypek et al., 2019). In this study, we have unraveled possible pathways in ovarian cells altered by radiation, thus providing insights on the mechanisms underlying the perturbed reproductive functions. More importantly, our findings suggested that ionizing radiation can interfere telomere and chromatin remodeling, leading to possible epigenetic changes. Further investigation is warranted to elucidate whether these induced modifications can lead to transgenerational reproductive impairments.

## Data Availability Statement

Sequencing data of transcriptome sequencing that support the findings of this study have been deposited in the NCBI BioProject database (https://www.ncbi.nlm.nih.gov/bioproject) with the BioProject accession code PRJNA730377.

## Ethics Statement

In accordance with the national legislation and the institutional requirements, the Human Research Ethics Committee of The University of Hong Kong waived the requirement for ethical approval and written informed consent for participants in this study.

## Author Contributions

RW, AW, WL, KWY, RK, and JC contributed to conception and design of the study. CL and YY performed the experiments. KL organized the database. TC, XL, and NT performed the bioinformatics and statistical analysis. KL, RW, and KNY wrote the manuscript. All authors contributed to manuscript revision, read, and approved the submitted version.

## Conflict of Interest

The authors declare that the research was conducted in the absence of any commercial or financial relationships that could be construed as a potential conflict of interest.

## Publisher’s Note

All claims expressed in this article are solely those of the authors and do not necessarily represent those of their affiliated organizations, or those of the publisher, the editors and the reviewers. Any product that may be evaluated in this article, or claim that may be made by its manufacturer, is not guaranteed or endorsed by the publisher.

## Footnotes

1
www.qiagen.com/ingenuity

2
https://www.ncbi.nlm.nih.gov/bioproject
