# Corrigendum: Low-Dose Radiation Can Cause Epigenetic Alterations Associated With Impairments in Both Male and Female Reproductive Cell

**DOI:** 10.3389/fgene.2022.945115

**Published:** 2022-06-15

**Authors:** Chi Tim Leung, Yi Yang, Kwan Ngok Yu, Nathan Tam, Ting Fung Chan, Xiao Lin, Richard Yuen Chong Kong, Jill Man Ying Chiu, Alice Sze Tsai Wong, Wing Yee Lui, Karen Wing Yee Yuen, Keng Po Lai, Rudolf Shiu Sun Wu

**Affiliations:** ^1^ Hong Kong Branch of the Southern Marine Science and Engineering Guangdong Laboratory, Hong Kong SAR, China; ^2^ Department of Chemistry, City University of Hong Kong, Hong Kong SAR, China; ^3^ School of Biological Sciences, The University of Hong Kong, Hong Kong SAR, China; ^4^ State Key Laboratory of Marine Pollution, City University of Hong Kong, Hong Kong SAR, China; ^5^ Department of Physics, City University of Hong Kong, Hong Kong SAR, China; ^6^ School of Life Sciences, Hong Kong Bioinformatics Centre, The Chinese University of Hong Kong, Hong Kong SAR, China; ^7^ Department of Biology, Hong Kong Baptist University, Hong Kong SAR, China; ^8^ Laboratory of Environmental Pollution and Integrative Omics, Guilin Medical University, Guilin, China; ^9^ Department of Science and Environmental Studies, The Education University of Hong Kong, Hong Kong SAR, China

**Keywords:** environmental radiation, epigenetic, reproductive impairments, testicular, ovarian

In the original article, there was an error. The origin of the cell lines was not stated clearly.

A correction has been made to **Materials and Methods**, *Ovarian and Testicular Cell Culture and Ionizing Radiation Exposure*, **Paragraph 1**:

Two human ovarian cancer cells (SKOV3 and COV434) and two mouse testicular germ cells (GC-1 and TM4) were cultured under the conditions described in **Supplementary Table S1**. Only human and mouse cell lines (not primary cells) were used, and these were purchased from an international company. In accordance with the national legislation and the institutional requirements, the Human Research Ethics Committee of The University of Hong Kong waived the requirement for ethical approval and written informed consent for participants in this study. The cells were cultured at 37°C under 95% air and 5% carbon dioxide. For the ionizing radiation exposure, the cells were seeded onto 6 well plate 1 day before exposure to 10 cGy of X-ray (320 kV, 2 mA) for 1 min (X-RAD 320 X-ray system).

The authors apologize for this error and state that this does not change the scientific conclusions of the article in any way. The original article has been updated.

